# Examining Relationships Among Alcohol Use Disorder, Child Caretaking, and Intimate Partner Violence in High-Risk Couples

**DOI:** 10.1007/s10896-024-00696-x

**Published:** 2024-04-12

**Authors:** Sarah T. Giff, Shannon R. Forkus, Andrea A. Massa, Jessica L. Brower, Amber M. Jarnecke, Julianne C. Flanagan

**Affiliations:** 1 Department of Psychiatry and Behavioral Sciences, Medical University of South Carolina, Charleston, South Carolina, USA; 2 Ralph H. Johnson VA Medical Center, Charleston, South Carolina, USA; 3 Department of Psychiatry, The University of North Carolina at Chapel Hill, Chapel Hill, NC, USA

**Keywords:** Intimate Partner Violence, Alcohol use Disorder, Caretaking, Parenting

## Abstract

**Purpose:**

Intimate partner violence (IPV) is a serious public health concern that is highly prevalent among couples with alcohol misuse. It is well-established that alcohol can exacerbate negative IPV outcomes; however, less is known about how hazardous alcohol consumption, combined with family composition, such as the presence of children in the home, may impact IPV in a dyadic context. The current study examined the separate and interactive roles of the couple’s caretaking status and alcohol use disorder (AUD) severity on psychological and physical IPV victimization.

**Methods:**

Secondary data were analyzed from 100 couples considered high risk due to reporting physical IPV and at least one partner meeting criteria for AUD. Multilevel mixture models were used to dyadically test how caretaking status and each partner’s AUD severity, separately and interactively, related to the couple’s psychological and physical IPV severity.

**Results:**

Caretaking status and one’s own AUD severity, when examined separately, were positively related to psychological and physical IPV victimization. One’s partner’s AUD severity was also related to severity of physical IPV victimization. There was no evidence of an interaction in this sample.

**Conclusions:**

Caretaking status played an important role in IPV victimization even when accounting for AUD in high-risk couples. Caretaking status and AUD did not interact; however, the significant main effects suggest an additive association, such that the combination of AUD severity and caretaking is more risky for IPV victimization than either factor alone. Findings highlight the importance of considering family composition and alcohol use behaviors on IPV risk.

## Introduction

Intimate partner violence (IPV) imposes significant individual and family costs (Bedi & Goddard, 2007; Jouriles et al., 2008; Lagdon et al., 2014; Riger et al., 2002) and is further complicated when one or both partners experiences alcohol use disorder (AUD). It is well-established that AUD and IPV frequently co-occur (e.g., Devries et al., 2014; Eckhardt et al., 2022). Indeed, AUD is a known precipitant to IPV (Leonard & Quigley, 2017) and is associated with more severe and frequent violence among couples (Graham et al., 2010; Leonard & Quigley, 2017; McKinney et al., 2010). Further, AUD is also a consequence of IPV (Øverup et al., 2015). Alcohol is often used in an effort to reduce psychological distress, consistent with negative reinforcement and self-medication models of alcohol use (Khantzian, 1997). Research also indicates that IPV events and patterns among couples are influenced by both the individual’s alcohol use behaviors *and* the partner’s alcohol use behaviors (Eckhardt et al., 2019), suggesting the need to study IPV and its risk factors in a dyadic context. Our understanding of dyadic processes involving both AUD and IPV continues to grow; however, less is known about the potential impact of family composition on IPV severity among couples with co-occurring AUD and IPV.

One specific aspect of family composition that is important, yet understudied, is the presence of children in the home, and how that may relate to IPV severity, especially when combined with caregiver who has AUD. Parenting or caretaking for children introduces a high level of responsibility in addition to financial and social stress. According to social stress theories, increased social pressures such as these are related to increased risk for IPV in relationships and AUD (Capaldi et al., 2012). Clarifying the combined impact of AUD with the presence of children in the home on IPV severity can provide useful information to facilitate the identification of high risk families and inform intervention efforts accordingly. Some epidemiological studies have identified elevated IPV rates for *individuals* with children compared to those without children. Most of these studies have focused on women’s increased victimization and men’s increased perpetration using samples of different sex couples and cis-gender participants (e.g., DeMaris et al., 2003; Jones et al., 1999). Bair-Merritt and colleagues (2008) identified that the risk of IPV victimization was even greater for women raising children who also reported alcohol use problems. It has yet to be determined if IPV victimization increases as well for men with children, as risk factors for IPV victimization among men are still relatively understudied (Spencer et al., 2019). Graham and colleagues (2021) found that living with children and greater drinking was related to victimization, perpetration, and bidirectional IPV among a large sample of participants across 14 countries. However, each of these studies investigated individuals rather than dyads. Most of these studies posit that raising children brings more stress and relational conflict, which increases risk for IPV.

Although most quantitative research to date suggests that caretaking for children may increase risk for IPV, qualitative studies indicate that having children may alternatively provide motivation for seeking treatment for IPV and maintaining alcohol recovery for parents more than non-parents. For example, Poole and Murphy (2019) found that fathers were more likely to engage in, attend, and complete an IPV intervention program compared to men who were not fathers, while men interviewed regarding core components of their identity and key motivations for change when entering IPV treatment reported their role as a father and a desire to improve relationships with their children as key elements driving their treatment engagement (e.g., Fox et al., 2002; Holt, 2015; Stanley et al., 2013). Similarly, mothers interviewed regarding motivations for help-seeking for IPV most frequently cited concerns about the effects of IPV on their children as their most important motivator (Randell et al., 2012). Qualitative interviews specifically with women coping with both AUD and IPV describe how motherhood played a primary role in how they thought about seeking help, but mothers also shared concerns about how caretaking introduced additional logistical barriers to accessing treatments, concerns about court involvement, and that drinking alcohol was one of their only strategies for coping with high levels of distress related to IPV (Bohrman et al., 2017; Rhodes et al., 2010; Seay et al., 2017). Overall, it seems that although caring for children may provide motivation to change some risky behaviors, the additional responsibilities of caretaking may also introduce practical barriers to change, and may further exacerbate social stress, which is a known contributor to both AUD and IPV (Capaldi et al., 2012; Esper & Furtado, 2013).

Although AUD is a known contributor to risk of IPV in relationships, little is known about how the presence of children in the home may relate to IPV in couples struggling with AUD. Thus, the primary aim of the current study is to examine the concurrent roles of presence/absence of children in the home and severity of AUD collected from a sample of couples with prior history of IPV and AUD, and to identify whether the presence of children and AUD severity interact to impact IPV victimization severity (psychological and physical) within a couple. We hypothesized that both having a child in the home and greater severity of AUD, separately, would be associated with greater severity of physical and psychological IPV victimization. We also expected that having a child at home and AUD severity would interact, such that greater AUD severity and having children in the home would be associated with greater physical and psychological IPV victimization severity.

## Method

### Participants

Participants included *N* = 100 romantic couples (*n* = 106 [53.0%] women; *n* = 92 different-sex couples, *n* = 8 same-sex couples) who took part in a larger preregistered randomized controlled trial (Flanagan et al., 2022) investigating the impacts of intranasal oxytocin on alcohol craving and laboratory-based IPV. To be eligible for the study, participants were required to be at least 18 years of age and to be in a committed relationship with their current partner for at least 6 months. In addition, at least one partner from each couple was required to meet DSM-5 (American Psychiatric Association, 2013) criteria for current AUD, as assessed by the Mini-International Neuropsychiatric Interview (MINI; Sheehan et al., 1998), and to report perpetration or victimization of at least one instance of physical IPV (e.g., pushing, hitting) on the Revised Conflict Tactics Scale (CTS-2; Straus et al., 1996). For the purposes of the larger trial, couples were excluded from the study if either partner obtained a score of 8 or higher on the Clinical Institute Withdrawal Assessment for Alcohol (CIWA-Ar; Sullivan et al., 1989) or if they endorsed severe and unilateral IPV in their current relationship on the CTS-2 to ensure participant safety. All couples who took part in the study were included in the current analyses.

Full sample descriptives have been reported previously (Flanagan et al., 2022). Participants were on average 35.43 (*SD* = 11.27) years of age and reported 14.13 (*SD* = 2.55) years of education. Participants reported an average relationship length of 89.23 (*SD* = 93.48) months. Most participants reported being unmarried but cohabiting (46.2%) or married (36.4%). The majority of participants identified as White (74.3%) or African American (19.1%), and most identified as non-Hispanic (93.5%). Nearly three quarters of participants were employed full-time (50.5%) or part-time (21.5%), and the average annual income was $69,706.95 (*SD* = $97,512.50). Approximately one-third (38.7%) of participants reported having biological children living with them, while 15.1% reported having non-biological children living with them. The majority of the sample (79.0%) met criteria for AUD, and both partners had AUD in 58% of couples in the sample. On average, participants reported 47.24% drinking days (*SD* = 33.95) and 25.09% (*SD* = 31.72) heavy drinking days (≥ 4 drinks for women, ≥ 5 drinks for men) out of the past 60 days.

### Procedures

Couples were recruited primarily via online (i.e., Craigslist, Facebook) and print advertisements. Each partner completed a telephone screening call to determine preliminary eligibility for the study. Couples then completed informed consent and a full baseline assessment. Those who were eligible were invited to participate in a single laboratory session. The current study used data from the baseline assessment only, and full study procedures are published elsewhere (Flanagan et al., 2022). Due to the COVID-19 pandemic, 16 couples completed informed consent and the baseline assessment via HIPAA-compliant telehealth platforms, while the remaining 84 couples completed all study procedures in person. All study procedures were approved by the local Institutional Review Board.

### Measures

#### Caretaking Status

Participants were asked to indicate the number of biological and non-biological children that were currently living with them in the home. These data were used to create a dichotomous variable indicating presence or absence of caretaking status for each couple. Eight couples reported discrepancies or had missing data from one partner. Among these eight couples, if at least one member of the couple reported a child living at home and the couple was married or cohabitating, they were coded as having children at home (*n* = 4), and if there was a discrepancy and the couple was not married or cohabitating, they were coded as not having children at home (*n* = 4). Almost half (*n* = 44) of the couples reported having at least one child in the home (range of 1 to 6 children). Children ranged in age from approximately 2 months to 24 years.

#### AUD Severity

The 10-item Alcohol Use Disorders Identification Test (AUDIT; Saunders et al., 1993) assesses past-year problematic patterns of alcohol use. Total scores range from 0 to 40, with higher scores indicating more alcohol misuse. Sample items include “How many drinks containing alcohol do you have on a typical day when you are drinking” and “Have you or someone else been injured as a result of your drinking?” The AUDIT is widely used and has evidenced strong construct validity and internal reliability in a range of samples (Babor et al., 2001; de Meneses-Gaya et al., 2009; Saunders et al., 1993). The internal consistency in the current sample is α = 0.89. The average AUDIT score was 10.98 (*SD* = 7.63), falling in a range suggesting hazardous or harmful alcohol consumption.

#### Intimate Partner Violence

The 78-item Revised Conflict Tactics Scale (CTS-2; Straus et al., 1996) assesses the frequency of victimization and perpetration of physical, psychological, and sexual IPV, as well as the use of negotiation and reasoning to handle relationship conflicts, in participants’ current relationships over the past 6 months. The CTS2 is considered the “gold standard” measure of IPV and has demonstrated strong internal consistency as well as construct and discriminant validity (Chapman & Gillespie, 2019; Straus et al., 1996). The 12-item physical IPV (e.g., “Have you pushed or shoved your partner?”) and 8-item psychological IPV (e.g., “Have you insulted or sworn at your partner”) victimization subscales were used in the current analyses. Response options range from 0 (never in the past 6 months) to 6 (more than 20 times in the past 6 months). Three response options that depict a range (3 = 3–5 times in the past 6 months, 4 = 6–10 times, 5 = 11–20 times) were recoded to reflect the midpoint of that range. In addition, the response option “more than 20 times in the past 6 months” was recoded as 25. Thus, total scores could range from 0 to 300 for physical IPV and 0-200 for psychological IPV. Internal reliabilities for the scales were good with α’s between 0.74 and 0.86. Consistent with previous studies and to minimize the effect of underreporting, a maximum scoring method was used (Taft et al., 2010, 2016). This method involves using the higher score between the participant’s own report of IPV victimization or their partner’s report of IPV perpetration for each item when calculating each participant’s IPV subscale scores. Average maximum scores of IPV were 42.91 (*SD* = 32.66) for psychological IPV victimization and 33.05 (*SD* = 32.76) for physical IPV victimization.

### Analytic Plan

Preliminary analyses were conducted to inspect means, standard deviations, and bivariate correlations among variables. Due to the use of secondary data, a power analysis was not conducted; rather, recommended procedures for reporting statistics were used (Dziak et al., 2020). All primary analyses were conducted within a multilevel mixture modeling framework using SPSS version 26. Actor-partner interdependence modeling was used, as this approach accounts for interdependence of data within dyads and allowed us to examine the effects of each partner’s alcohol use severity on IPV outcomes (Kenney et al., 2006). Level-1 predictors included grand-mean centered individual and partner AUDIT scores, while caretaking status of the couple (effects coded as −0.5 for no children at home and +0.5 for children at home) was included as a Level-2 couple variable. Two cross-level interaction terms were created, one for each centered actor and partner AUDIT score multiplied by effects-coded caretaking status. Associations of these predictors were assessed separately for the psychological IPV and physical IPV victimization. We planned to probe any interactions to better understand the directions of effects, and to remove any non-significant interactions to preserve power. Of note, all analyses were also conducted controlling for gender, which was not significant and did not change the significance or direction of any effects, so it was removed from final models to preserve power.

## Results

Means, standard deviations, and correlations among primary variables are shown in Table 1. Caretaking status was positively correlated with psychological (*r* = .19, *p* = .008) and physical (*r* = .17, *p* = .015) IPV victimization, and was negatively correlated with one’s own (*r* = − .18, *p* = .011) and one’s partner’s (*r* = − .18, *p* = .011) AUD severity. As shown in Table 2, results from the model examining psychological IPV victimization indicate that caretaking status (*b* = 17.57, *p* = .005), one’s own AUD severity (*b* = 1.72, *p* < .001), and one’s partner’s AUD severity (*b* = 0.64, *p* = .050) were positively related to greater psychological IPV victimization severity. The interactions of AUD severity and caretaking status were not related to psychological IPV victimization. Physical IPV victimization followed the same pattern, as caretaking status (*b* = 15.94, *p* = .004), one’s own AUD severity (*b* = 0.85, *p* = .010) and one’s partner’s AUD severity (*b* = 0.76, *p* = .020) were all positively related to physical IPV victimization severity. Interactions of AUD severity by caretaking status were not significantly associated with physical IPV victimization severity.

Because no statistically significant interactions emerged, subsequent post-hoc analyses removing the interactions were conducted for parsimony and to improve power. As shown in Table 3, when examining psychological IPV victimization severity, findings were somewhat consistent with the model that included the interaction terms. Caretaking status (*b* = 16.59, *p* = .007) and one’s own AUD severity (*b* = 1.02, *p* < .001) were positively related to psychological IPV victimization, but partner’s AUD severity was no longer related to psychological IPV victimization. For physical IPV victimization, the results remained the same as the model with the interactions terms; caretaking status (*b* = 15.23, *p* = .006), one’s own AUD severity (*b* = 0.78, *p* = .009), and one’s partner’s AUD severity (*b* = 0.63, *p* = .035) were positively related to physical IPV victimization severity.

## Discussion

This study sought to identify how children in the home and AUD severity separately, and in combination, related to physical and psychological IPV victimization in couples with physical IPV and at least one member who had AUD. Our a priori hypotheses were partially supported, as we found that a couple’s caretaking status and one’s own severity of AUD were positively related to psychological IPV victimization, while the same variables in addition to one’s partner’s AUD severity were positively related to physical IPV victimization. However, there was no evidence in our sample of an interaction between AUD severity and the presence of children in the home.

The main effects demonstrating that one’s own and/or one’s partner’s AUD problems were related to both psychological and physical IPV victimization are congruent with decades of research identifying links between alcohol problems and IPV (Devries et al., 2014; Foran & O’Leary, 2008).One notable explanation for the association between one’s own alcohol use and associated IPV victimization is the self-medication model which proposes that individuals engage in alcohol use to cope with chronic stressors such as relationship violence (Øverup et al., 2015). The association between one’s partner’s AUD and victimization may be due to alcohol’s influence on cognitive processes. Specifically, alcohol can interfere with higher-order cognitive processes (Steele & Josephs, 1990), including decision-making, impulse control, and emotion regulation, limiting the ability to process and effectively respond to environmental cues (e.g., responding with aggression) thereby increasing risk for IPV (Crane et al., 2016). Also, individuals with AUD are more likely to have a partner who also uses alcohol, which increases risk for relational conflict and IPV perpetration (Devries et al., 2014; Flanagan et al., 2023; Muyingo et al., 2020); however, after accounting for caretaking status, we found that one’s partner’s alcohol problems were only associated with physical, but not psychological IPV in our final, more parsimonious model. Our findings corroborate past research on AUD and IPV victimization, and extends findings by examining associations within a dyadic context and among IPV couples with AUD.

The main effects demonstrating that a couple’s caretaking status was positively related to both psychological and physical IPV victimization severity represents a novel finding, especially in this complex population. Several prior studies have investigated how having children may relate to mothers’ risk of IPV victimization (DeMaris et al., 2003; Jones et al., 1999; Nash et al., 2022), and one prior study identified that both having children and reporting problems with alcohol use was related to mothers’ greater risk of IPV victimization (Bair-Merritt et al., 2008). However, this is the first known study to conduct a dyadic investigation of the association between children in the home using maximum scoring to account for under-reporting, and to examine both partners simultaneously. Through the social stress theory lens, it is possible that the additional responsibility of taking care of children in the home increased stress, thereby increasing AUD severity and IPV. Some qualitative research also suggests that sharing children may introduce unique barriers to ending unhealthy relationships characterized by greater IPV for both for child-focused (i.e. not wanting to separate a child and caregiver) and logistical (i.e. financial, safety) reasons (Bohrman et al., 2017). Of note, we also did not observe any gender differences in the relationship between AUD and caretaking and IPV. Much of our existing knowledge about child-rearing and AUD and IPV often focuses either on exclusively mothers or fathers, and our results indicate that these results are important regardless of gender.

Despite finding significant main effects for both AUD and children in the home in relation to both physical and psychological IPV victimization, we did not identify any interactions between the two variables. This is consistent with one study asking a similar question in a larger epidemiological sample (Graham et al., 2021), which also found significant main effects but no interaction between children at home and AUD severity in relation to IPV. Although these factors did not interact, the significant main effects suggest that when both variables are included in the same model that there is an additive association, such that the combination of AUD severity *and* having children at home is more risky for IPV than either one of these factors in a family alone. Although some qualitative studies suggest that having children in the home may provide more motivation to seek treatment for both IPV and AUD (e.g., Fox et al., 2002; Holt, 2015; Poole & Murphy, 2019; Stanley et al., 2013), it is also possible that caretaking for children presents logistical barriers for treatment engagement, retention, and completion, limiting effectiveness of such treatment.

In this dyadic analysis, although we consistently found that one’s own AUD severity was related to both physical and psychological IPV victimization, we did not find that one’s partner’s AUD severity was related to psychological IPV victimization when only testing main effects (without the interaction). These results suggest that greater AUD severity among each caretaker in the same household may increase the likelihood of conflict escalation to more severe physical IPV but may not operate in the same pattern for psychological IPV. Alternatively, it is also likely that when caretaking status is accounted for in the model, the effects of partner alcohol use is not as relevant to psychological IPV as one’s own alcohol use, especially given ample literature demonstrating contribution of partner’s alcohol use to IPV (e.g., Cafferky et al., 2018; Thompson & Kingree, 2006).

Although this project has many strengths, such as using a maximum reporting score of IPV victimization to reduce reporter bias, using dyadic analyses to account for the contribution of both partners alcohol use to IPV, including an AUD diagnostic sample, and including both mixed and same-sex couple relationships, there are several limitations. First, these data are cross-sectional and causality cannot be inferred. As noted by some participants in qualitative studies (e.g., Baker & Carson, 1999), IPV and AUD severity may be cyclically related, as individuals may continue problematic drinking as a way to cope with IPV-related distress, and such drinking likely exacerbates the potential for conflict and IPV. The cross-sectional nature of the study cannot establish temporal associations, and a larger sample size would be necessary to test other control variables that may be important, such as demographic or contextual variables about the individuals, the couple, or the children in the home. In addition, this study only examined caretaking status as a dichotomous variable, and more nuanced information, such as the age, gender, and number of children may shed more light on these families.

Understanding the family context for IPV is important, as it is well-demonstrated that childhood exposure to adult IPV can have wide-ranging negative impacts for children’s social and emotional development (Fritz & Roy, 2022). Similarly, children growing up in homes where parents experience more severe AUD also demonstrate more risk for negative outcomes, such as greater behavioral problems and higher rates of substance use disorders in adolescence (Straussner & Fewell, 2018). As such, the context of both IPV and AUD likely creates an environment in which children are doubly disadvantaged (see review by Klostermann & Kelley, 2009). For example, Stover and colleauges (2013) identified that fathers with co-occurring IPV and substance use problems reported more negative parenting and coparenting behaviors and more child social and behavioral problems compared to community controls without IPV and substance use problems.

The cascade of challenges that may stem from IPV and AUD justifies the need to address these issues in families who may continue to convey intergenerational risk. As such, our findings that caretaking for children independently relates to increased severity of IPV, even when accounting for each partner’s AUD, draws attention to a higher-risk population for treatment. Those looking to improve couples-based AUD and IPV treatments may consider changes to both the structure and content of treatment. Structural changes may include offering childcare and more flexible scheduling options (e.g., telehealth, evening appointments) to accommodate patients with caretaking responsibilities (e.g., Stover & Morgos, 2013). These types of accommodations may enhance patients’ ability to attend and complete treatment and reduce barriers to accessing care. Content covered in treatment may also specifically address how IPV and AUD may operate in the home, such as assessing safety and risk for children who may be exposed to IPV and/or heavy drinking and including healthy parenting and caretaking components. Long term, we also know that children exposed to IPV, and specifically bidirectional IPV (e.g., Eriksson & Mazerolle, 2015), grow up with an increased likelihood of using IPV themselves in future relationships. It is therefore extremely important that future research continues to investigate ways to support caretakers and children to prevent these problems from carrying forward to future generations. Our lack of differences in our findings across gender also reinforces that these services should be available to all patients, regardless of gender, which is important as many treatment settings may focus parenting programming exclusively on women. Dyadic or partner-included interventions may be especially important to address these issues so that partners can learn ways to address AUD while also learning healthy emotion regulation and conflict resolution skills to address IPV, as these issues are so inter-related.

## Figures and Tables

**Table 1:** Means, standard deviations, and correlations of primary variables

Variable	M (SD)	1	2	3	4	5	6
1.Caretaking Status	0.44 (0.50)	—					
2. Psychological IPV - Actor	42.6 (32.7)	0.19**	—				
3. Psychological IPV - Partner	42.6 (32.7)	0.19**	0.78**	—			
4. Physical IPV- Actor	33.1 (32.8)	0.17*	0.82**	0.63**	—		
5. Physical IPV - Partner	33.1 (32.8)	0.17*	0.63**	0.82**	0.41*	—	
6. AUDIT - Actor	11.0 (7.6)	−0.18**	0.20**	0.08	0.14**	0.12	—
7. AUDIT - Partner	11.0 (7.6)	−0.18**	0.08	0.20**	0.12	0.14*	0.02

Note. IPV = Interpersonal Violence; AUDIT = Alcohol Use Disorders Identification Test

* = *p* < .05.

** = *p* ≤ .01.

*** = *p* ≤ .001

**Table 2:** Multilevel mixture models of caretaking status, AUDIT, and AUDIT x caretaking interaction predicting psychological and physical interpersonal violence (IPV)

Model and Variables	*b*	SE	*p*-value	95% Confidence Interval of Unstandardized Parameters	
				Lower Bound	Upper Bound
Psychological IPV					
**Intercept**	**44.55**	**3.04**	**< 0.001**	**38.53**	**50.57**
**Caretaking Status**	**17.57**	**6.07**	**0.005**	**5.53**	**29.62**
**AUDIT– Actor**	**1.72**	**0.32**	**< 0.001**	**0.53**	**1.81**
**AUDIT – Partner**	**0.64**	**0.32**	**0.050**	**−0.00**	**1.27**
Caretaking x AUDIT Actor Interaction	0.70	0.64	0.279	−0.57	1.97
Caretaking x AUDIT Partner Interaction	0.60	0.64	0.350	−0.67	1.88
Physical IPV					
**Intercept**	**34.56**	**2.71**	**< 0.001**	**29.27**	**40.03**
**Caretaking Status**	**15.94**	**5.42**	**0.004**	**5.19**	**26.70**
**AUDIT– Actor**	**0.85**	**0.32**	**0.010**	**0.21**	**1.49**
**AUDIT – Partner**	**0.76**	**0.32**	**0.020**	**0.12**	**1.40**
Caretaking x AUDIT Actor Interaction	0.30	0.65	0.646	−0.98	1.58
Caretaking x AUDIT Partner Interaction	0.65	0.65	0.321	−0.63	1.93

Note. Bolded are significant predictors; AUDIT = Alcohol Use Disorders Identification Test

**Table 3:** Multilevel mixture models of caretaking status and AUDIT predicting psychological and physical interpersonal violence (IPV)

Model and Variables	*b*	SE	*p*-value	95% Confidence Interval of Unstandardized Parameters	
				Lower Bound	Upper Bound
Psychological IPV					
**Intercept**	**43.61**	**2.92**	**< 0.001**	**37.81**	**49.40**
**Caretaking Status**	**16.59**	**6.04**	**0.007**	**4.62**	**28.56**
**AUDIT– Actor**	**1.02**	**0.29**	**< 0.001**	**0.44**	**1.60**
AUDIT – Partner	0.51	0.29	0.087	−0.08	1.09
Physical IPV					
**Intercept**	**33.96**	**2.60**	**< 0.001**	**28.80**	**39.13**
**Caretaking Status**	**15.23**	**5.38**	**0.006**	**4.55**	**25.90**
**AUDIT– Actor**	**0.78**	**0.30**	**0.009**	**0.19**	**1.37**
**AUDIT – Partner**	**0.63**	**0.30**	**0.035**	**0.04**	**1.21**

Note. Bolded are significant predictors; AUDIT = Alcohol Use Disorders Identification Test
